# Spatially-offset optical coherence tomography (SO-OCT) for high dynamic range biological imaging at depth

**DOI:** 10.1364/BOE.607448

**Published:** 2026-09-16

**Authors:** Emi C. V. Hughes, Ralf Mouthaan, Freja Høier, Gavrielle R. Untracht, Peter E. Andersen, Kishan Dholakia

**Affiliations:** 1 Centre of Light for Life, School of Biological Sciences, Adelaide University, Adelaide, SA 5005, Australia; 2 Department of Health Technology, Technical University of Denmark, Ørsteds Pl. 345C, 2800 Kongens Lyngby, Denmark; 3 SUPA, School of Physics and Astronomy, University of St Andrews, St Andrews KY16 9SS, UK

## Abstract

Optical coherence tomography (OCT) in highly scattering media is limited by strong backscattering from superficial layers, which reduces sensitivity to weaker signals from deeper regions. Spatially-offset OCT (SO-OCT) addresses this limitation by introducing laterally displaced illumination and detection paths, thereby preferentially sampling photons originating from increased depths. Here, we demonstrate a high dynamic range imaging approach combining acquisitions at multiple spatial offsets and exposure times, using a tailored "weighted root-sum-square" merging algorithm. The resulting images exhibit enhanced contrast at depth without compromising structural fidelity in shallow regions. We achieve up to a tenfold improvement in contrast-to-noise ratio (CNR) in optical phantoms and a threefold improvement in *ex vivo* biological tissues compared to conventional OCT without any spatial offset. These results highlight the potential of SO-OCT combined with image merging to improve depth-resolved imaging and extend the practical dynamic range of OCT in biologically relevant scattering media.

## 1. Introduction

Optical coherence tomography (OCT) is a noninvasive, label-free imaging modality that provides volumetric, depth-resolved imaging [1]. It is widely used in biomedical applications such as clinical ophthalmology [2], neuroimaging [3], dermatology [4,5], dentistry [6] and cardiology [7]. Spectral-domain OCT (SD-OCT) acquires spectral interferometric signals such that information from a range of imaging depths is captured simultaneously, enabling rapid high-resolution volumetric imaging. The effective imaging depth of SD-OCT is limited by several factors, including depth-dependent signal attenuation, illumination beam quality, collection efficiency, and dynamic range of the detection system. In particular, signals from shallow layers often dominate weaker signals from deeper layers due to their higher intensity [8]. Given the limited dynamic range of the detector, this large disparity in signal strength can limit the detection of weaker signals from deeper regions, thereby reducing the effective imaging depth of SD-OCT, particularly in biological tissues, which are often highly scattering.

Various approaches have been proposed to mitigate this limitation, including modifications to the system geometry [9,10]. In our previous work, we introduced spatially-offset OCT (SO-OCT), which uses parallel illumination and collection beams that are laterally offset from one another [11]. This spatial offset acts to suppress near-axis light originating from the surface and enables preferential collection of light from deeper layers, thereby improving depth sensitivity. Consequently, small spatial offsets combined with short exposure times can effectively sample superficial layers, whereas larger spatial offsets combined with longer exposure times enable probing of deeper structures without introducing saturation artifacts, hence revealing features that are not visible using conventional OCT. Importantly, this is achieved using a straightforward modification to the imaging setup, without requiring a longer excitation wavelength, thereby avoiding inherent resolution trade-offs [12].

In this work, we investigate SO-OCT in detail and demonstrate how each region of the sample may be optimally exposed in at least one combination of spatial offset and exposure time. Consequently, a simple approach inspired by high dynamic range (HDR) photography [13] can subsequently be used to merge individual images and extend the effective dynamic range of the system. A selection of optical phantoms and highly scattering *ex vivo* biological samples are considered to systematically demonstrate that this technique yields a merged image revealing features at depth while maintaining high image quality at the surface. Substantial contrast-to-noise ratio (CNR) enhancement is observed at depth, with up to a tenfold improvement in an optical phantom, and threefold improvement in various types of mouse tissue.

## 2. Methods

### 2.1. Experimental setup

Figure 1(a) illustrates a simplified schematic of the SO-OCT system. A superluminescent diode (SLD; S-850-G-I-20, Superlum Broadlighters, Ireland; central wavelength of 841 nm, bandwidth of 31.3 nm), with an output power of 10.8 mW, is collimated and split by a beam splitter (*B*_1_; 90% power in sample arm, 10% power in reference arm). In the sample arm, the beam is scanned by a galvanometer (G; GVS011/M, Thorlabs, USA), expanded by a 4f lens pair, and focused onto the sample with a low-NA objective (Obj; LSM 03-BB, Thorlabs, USA). The sample is illuminated with 2.9 mW of optical power. Back-scattered light arising from refractive index mismatches within the sample returns through the same optical path. It is then de-scanned by the galvanometer, and separated from the illumination light by a beam splitter (*B*_2_; reflection 45%, transmission 55%). The light is refocused by lens (*L*_2_), and passes through a beam splitter (*B*_4_; reflection 45%, transmission 55%). In the reference arm, light passes through a neutral-density filter (ND; optical density adjusted to attenuate reference arm signal that is tilted to avoid spurious reflections) and undergoes a double-pass through a dispersion compensation unit (DC; LSM03DC, Thorlabs, USA) to match dispersion due to the objective. The beam then propogates through a beam splitter (*B*_3_; reflection 45%, transmission 55%), reflects from a mirror (M), and is focused by a lens (*L*_1_) before being directed to a beam splitter (*B*_4_) where it recombines with the sample arm. The combined signal is focused through a 50 *μ*m-diameter pinhole (PH) for spatial filtering. As the pinhole is conjugate to the sample plane, it allows for effective suppression of signal outside the region of interest. The combined beams are collimated by an achromatic doublet (*L*_3_), and are incident on a diffraction grating (GR; 1,100 lines per mm). The first diffraction order is imaged by a line-scan camera (Aviiva EM1 line-scan camera, e2v technologies, UK; maximum line rate 56 kHz) for spectral detection. The system provides an axial resolution of 10 *μ*m and a lateral resolution of 18 *μ*m. The system exhibits a 6 dB roll-off of 1.75 mm, measured by translating a mirror in the reference arm and recording the decay in the interferometric signal amplitude with depth, as shown in Fig. 1(b). The axial focus is positioned at a depth of 0.5 mm, leading to the corresponding intensity peak observed at this depth.

The Mach-Zehnder interferometer configuration decouples the reference arm from the sample arm. This facilitates independent implementation of a spatial offset in the sample arm without affecting the reference arm and allows for the collected optical powers in the two arms to be adjusted independently. The spatial offset is introduced by laterally displacing lens *L*_2_ and is defined as the separation between the illumination and collection arms at the sample plane (denoted *S* in the inset of Fig. 1(a)). An auxiliary camera (Blackfly S GigE, Teledyne FLIR IIS, Canada, not shown in Fig. 1) was positioned in the sample plane to calibrate the relationship between the position of lens (*L*_2_) and the offset introduced in the sample plane.

**Fig. 1.**
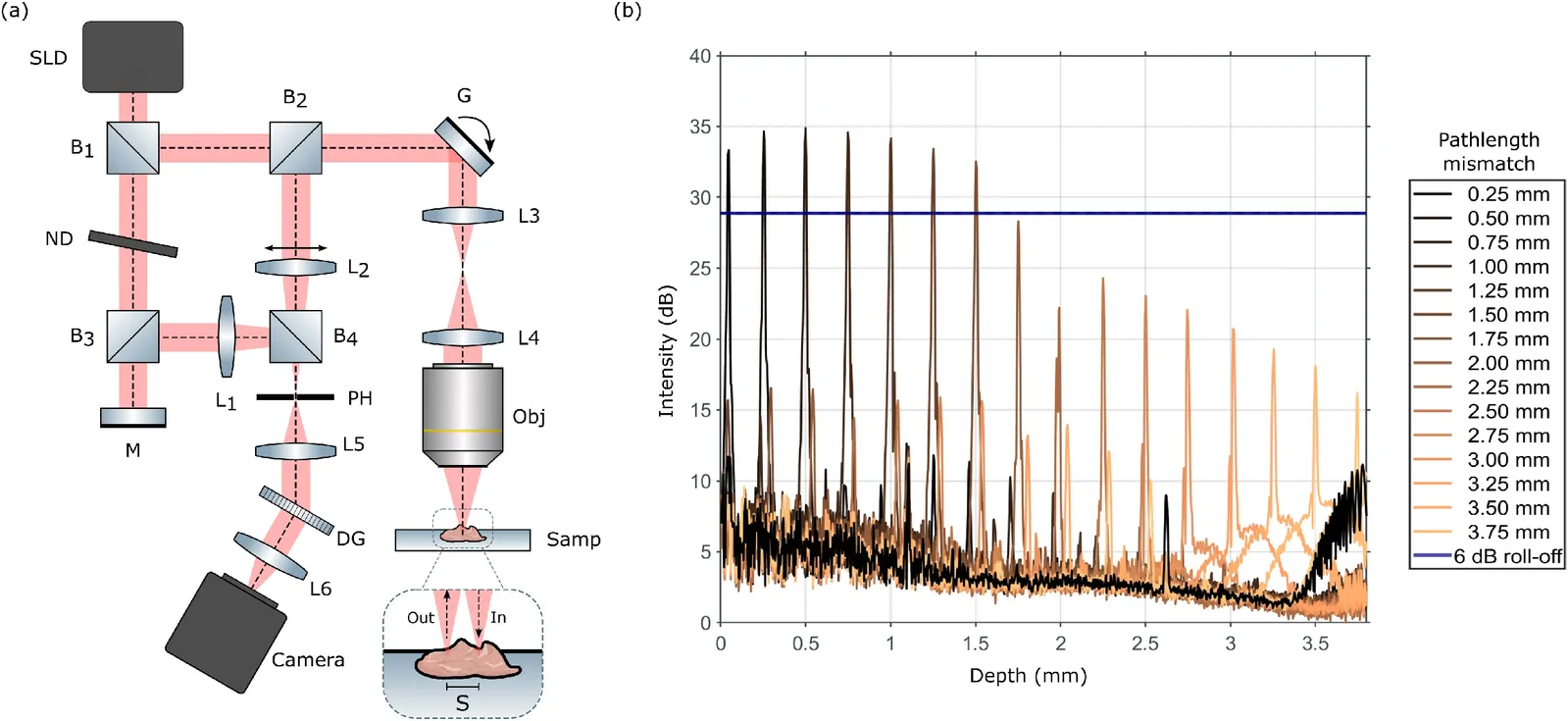
(a) Optical schematic for SO-OCT system. The inset indicates the spatial offset *S* between the illumination and collection beam. This spatial offset is introduced by translating a lens (*L*_2_) in the sample arm. Optical components are indicated as superluminescent diode (SLD), beam splitters $\left(B_{1}∼B_{4}\right)$ , x-axis galvanometer (G), lenses $\left(L_{1}∼L_{6}\right)$ , microscope objective (Obj), mirror (M), pinhole (PH), diffraction grating (DG), and line-scan camera (Camera). (b) Roll-off measurement of the SO-OCT system. The 6 dB roll-off is measured at a depth of 1.75 mm as indicated by the blue line.

### 2.2. Samples

Both phantoms and *ex vivo* biological tissue were used to evaluate the imaging performance of SO-OCT. By using samples with a variety of scattering and reflective properties, the effects of surface suppression and enhancement of CNR at depth were evaluated. Furthermore, the applicability of weighted root-sum-square (WRSS) was investigated across multiple samples.

The first phantom, which we call the "layered phantom", consists of a stack of microscope slide spacers (Sigma Aldrich, USA, thickness 120 *μ*m), and was used to evaluate the evolution of CNR with depth for given spatial offset values. A second phantom, which we call the "scattering phantom" consisted of a layer of acetate with white paint coated on the underside embedded in butter, where the butter acts as a strong scattering medium [11]. In-depth preparation procedures for all phantoms are noted in Supplement 1.

For validation of SO-OCT in biologically relevant samples, *ex vivo* Swiss albino mice tissues were dissected and fixed in 10% formalin for 4 weeks prior to imaging (n=5 mice). The samples include the head for ocular imaging, as well as the hind leg for imaging the paw pad and toe. All biological samples were obtained from scavenged animals already euthanized for purposes unrelated to this project. All animal procedures were conducted in accordance with the Australian Code of Practice for the Care and Use of Animals for Scientific Purposes.

### 2.3. Data acquisition

The interference signal between the sample arm and the reference arm was collected, as well as independent measurements from each arm. Background and autocorrelation contributions were suppressed by subtracting the reference-arm and sample-arm signals from the interference signal in the spectral domain. The resulting spectrum was resampled onto a uniformly spaced k-space grid. An inverse Fourier transform was then applied to yield an A-scan. The galvanometer scanner laterally scanned the beam, generating successive A-scans which were combined to form a B-scan image.

The maximum exposure time supported by the spectrometer camera was found to be 350 ms, where larger exposure times yielded excessive noise contributions. For moderately scattering samples, the maximum spatial offset was selected to probe the largest depth supported by the system (Fig. 1(b)), and the camera exposure was then set to avoid under- or over-exposure. For highly scattering samples, the maximum spatial offset was determined by setting the camera exposure time to 350 ms and increasing the spatial offset until the image acquired was not over-exposed. This large offset acquisition preferentially collects light from deep within the sample. Subsequent acquisitions were then performed using progressively smaller spatial offsets. In each case, the camera exposure time is adjusted such that the average surface signal is equalized across spatial offsets for each B-scan (additional details are provided in Supplement 1). These smaller offsets preferentially collect light from more superficial regions. The spatial offsets required to characterize the entire depth profile were found to strongly depend on the sample morphology. Exposure times for each sample and offset combination are provided in Supplement 1.

### 2.4. Merging of multiple spatially offset images

A comparison between several established image-merging algorithms is considered. These include: the incoherent average, whereby the magnitude of the OCT signal is first computed for each offset image and the resulting pixel intensities are then averaged; the coherent average, whereby the complex OCT signal is averaged across all offset images, then the magnitude is taken; the maximum projection, whereby the maximum intensity across all offsets is taken [14]; and the median projection, whereby the median intensity across all offsets is taken [15].

Additionally, we propose a bespoke image-merging method, named the "weighted root-sum-square algorithm (WRSS)". Here, a Gaussian smoothing kernel (standard deviation of 5 pixels) is applied to each individual offset image $I_{n}$ to obtain a weighting mask $M_{n}$ that describes what region is yielding a strong signal for a given offset. A small offset and short exposure time typically yields a mask with larger weightings in the surface region. Conversely, a large offset and long exposure time yields a mask with larger weightings in deeper regions. The merged image $I_{\text{merged}}$ is formed by calculating the weighted root-sum-square image,

(1) $$I_{\text{merged}}=\sqrt{\sum_{i}{\left(M_{i}I_{i}\right)}^{2}}.$$

All images are converted to linear intensity units before any calculations are performed. This approach allows any number of images to be merged, without requiring knowledge of the scattering parameters of the tissue or spatial offsets and exposure times used. Consistent signal contributions are emphasized, while noise is suppressed. As a result, this method provides contrast enhancement at depth without compromising superficial features.

An example calculation is provided in Fig. 2 for the mouse toe tissue. Five images, acquired at different spatial offsets and with different exposure times, are processed to obtain weighting masks and then combined to form a merged image. A general trend is observed in which larger offsets provide signal from deeper regions. Individual offsets also can preferentially detect localized features. For example, the third offset gives a mask that highlights a single scatterer at medium depth. This offset preferentially collects light from a directional Mie scatterer, as discussed by Untracht et al. [11], and it can be seen that the WRSS algorithm provides this with an appropriate weighting such that this scatterer is not over- or under-represented in the merged image. Compared with a direct weighted sum, the WRSS approach provides a more balanced combination of signal contributions across the entire depth range, whereas the weighted-sum approach reduces contrast from superficial layers. Images merged with each method are compared in Supplement 1.

**Fig. 2.**
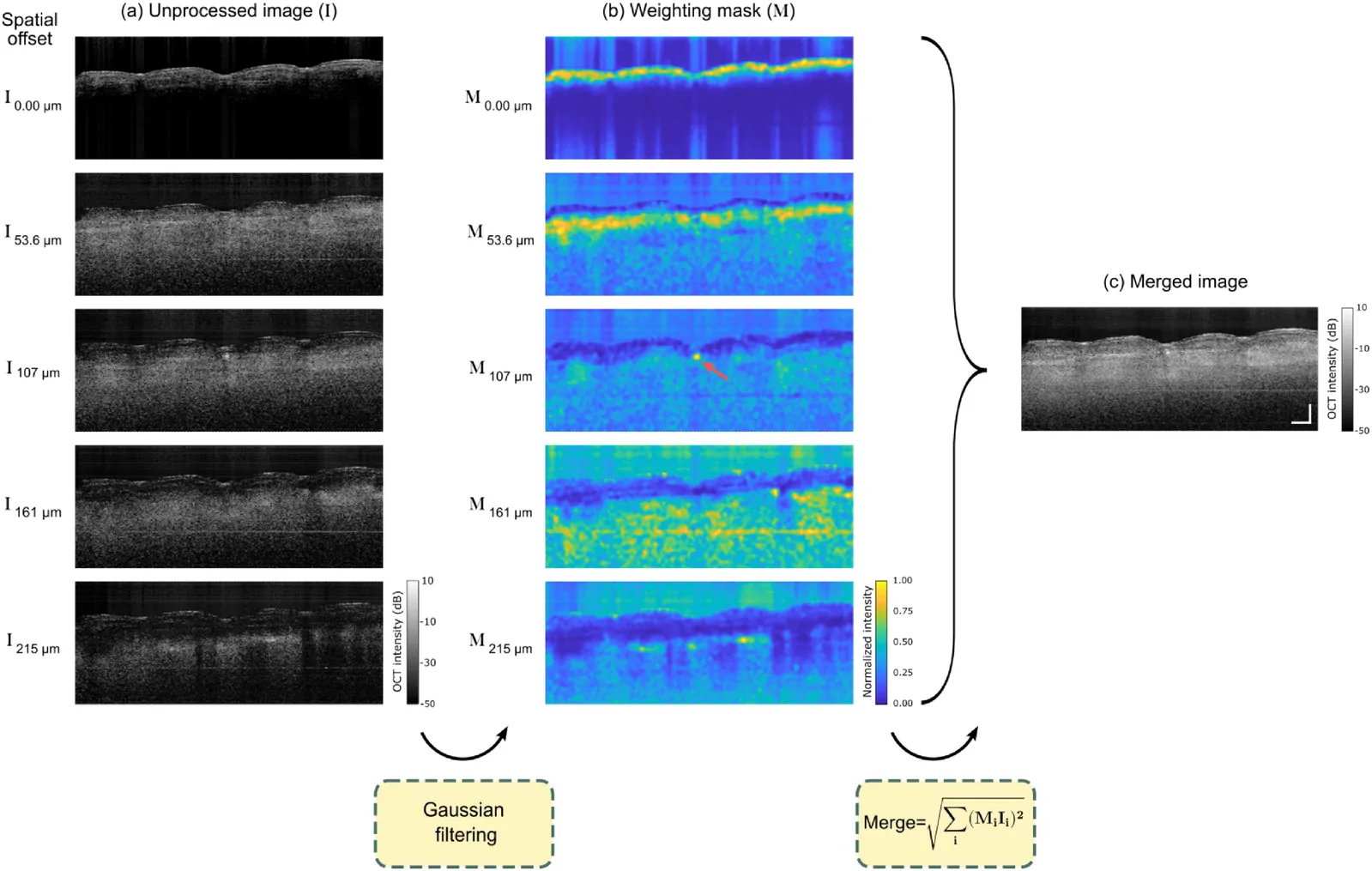
Visualization of the weighted root-sum-square (WRSS) process on mouse toe tissue. (a) The unprocessed images are denoted *I*. Each spatial offset exhibits a preferential imaging depth; smaller offsets provide the strongest contrast in superficial layers, whereas larger offsets enhance contrast at greater depths. The unprocessed images are Gaussian-filtered to compute a smoothed weighting mask. (b) The resulting mask, denoted *M*, is used to estimate the local signal contribution. For visualization purposes, each mask is normalized in this figure, with intensity mapped according to the color bar. The red arrow points to the single scatterer medium depth, which is only visible for a medium spatial offset. (c) The mask is then applied to generate a stack of weighted images, which are combined through a WRSS operation to produce a final image with improved contrast throughout the tissue volume. Scale bar: 500 *μ*m.

### 2.5. Image quality metrics

To enable quantitative comparison of image quality, we define the CNR metric,

(2) $$\text{CNR}=\frac{\left|\mu_{\text{signal}}-\mu_{\text{background}}\right|}{\sqrt{\left(\sigma_{\text{signal}}^{2}+\sigma_{\text{background}}^{2}\right)}},$$

in addition to the CNR improvement factor,

(3) $$\text{Improvement factor}=\frac{CNR_{\text{offset}}}{{\text{CNR}}_{\text{no offset}}},$$

where $\mu_{\text{signal}}$ and $\mu_{\text{background}}$ are the mean intensities of the region of interest (ROI) and background, respectively. Their corresponding standard deviations are expressed as $\sigma_{\text{signal}}$ and $\sigma_{\text{background}}$ .

In addition to CNR, the merging algorithms were compared in terms of signal-to-background ratio (SBR), which we define as,

(4) $$\text{SBR}=\frac{\mu_{\text{signal}}}{\mu_{\text{background}}}.$$

## 3. Results

### 3.1. Comparison of merging algorithms

The performance of the different merging algorithms is first qualitatively evaluated, with results on mouse toe tissue shown in Fig. 3. The incoherent average algorithm (Fig. 3(a)) yields an image with a large amount of background noise where fine morphological detail is washed out. This noise floor is reduced when using the coherent average (Fig. 3(b)), but fine detail is not yet clearly visible. The maximum projection algorithm (Fig. 3(c)) improves the visibility of deep tissue morphology, however, the superficial skin layers appear blurred, reducing the distinction between individual layers. Furthermore, the elevated noise floor and increased blur at extreme depths provide little additional structural information despite the higher signal intensity. Median projection (Fig. 3(d)) shows the lowest overall signal intensity, resulting in reduced contrast at superficial skin layers. However, morphological information is well preserved in deeper tissue layers. In contrast, the proposed WRSS projection (Fig. 3(e)) provides higher contrast in superficial layers than the maximum projection, without compromising on signal intensity, enabling for the clear separation of skin layers. At greater depths, sufficient signal and contrast is retained to preserve the visibility of deeper structures. The WRSS projection is used in the remainder of this paper. The CNR and SBR values were calculated for each image merging algorithm presented in Fig. 3, as shown in Table 1.

**Fig. 3.**
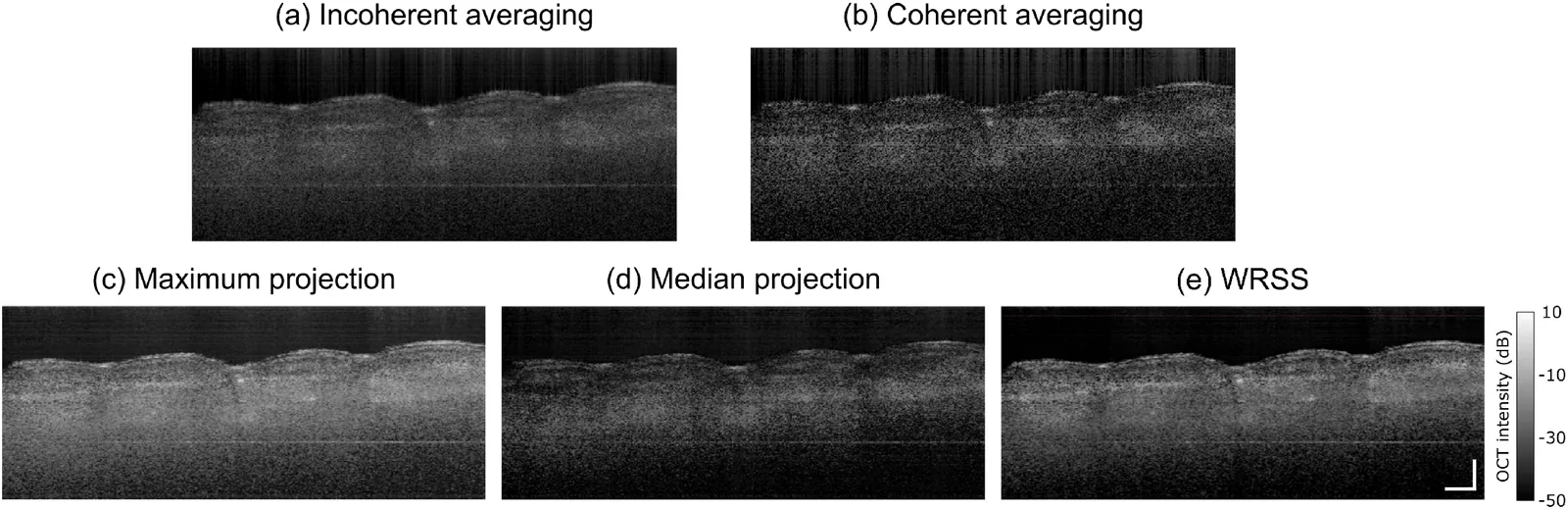
Comparison of image-merging methods applied to SO-OCT images acquired of mouse toe tissue; (a) coherent averaging, (b) incoherent averaging, (c) maximum projection, (d) median projection and (e) weighted root-sum-square (WRSS) projection. All images are displayed using the same intensity scale. Scale bar: 500 *μ*m

**Table 1.** Calculated CNR and SBR values for each image-merging algorithm. The WRSS algorithm achieved the highest CNR and SBR among the image algorithms evaluated.

	Incoherent average	Coherent average	Maximum projection	Median projection	WRSS projection

CNR	1.67	0.92	1.85	1.10	**1.88**
SBR	1.31	1.50	2.67	4.09	**5.44**

### 3.2. Layered phantom

In the first instance, we consider the layered phantom and evaluate the CNR at each layer for given spatial offset values. Figure 4(a) shows B-scan images acquired at different spatial offsets. As the offset was increased, the spectrometer camera exposure time was extended to ensure a consistent average surface intensity ( Supplement 1). Otherwise hidden, deeper layers become evident at larger offsets, increasing the effective imaging depth from approximately 2.0 mm to 3.5 mm when comparing the zero offset and the maximum offset (SO=16.1 *μ*m).

**Fig. 4.**
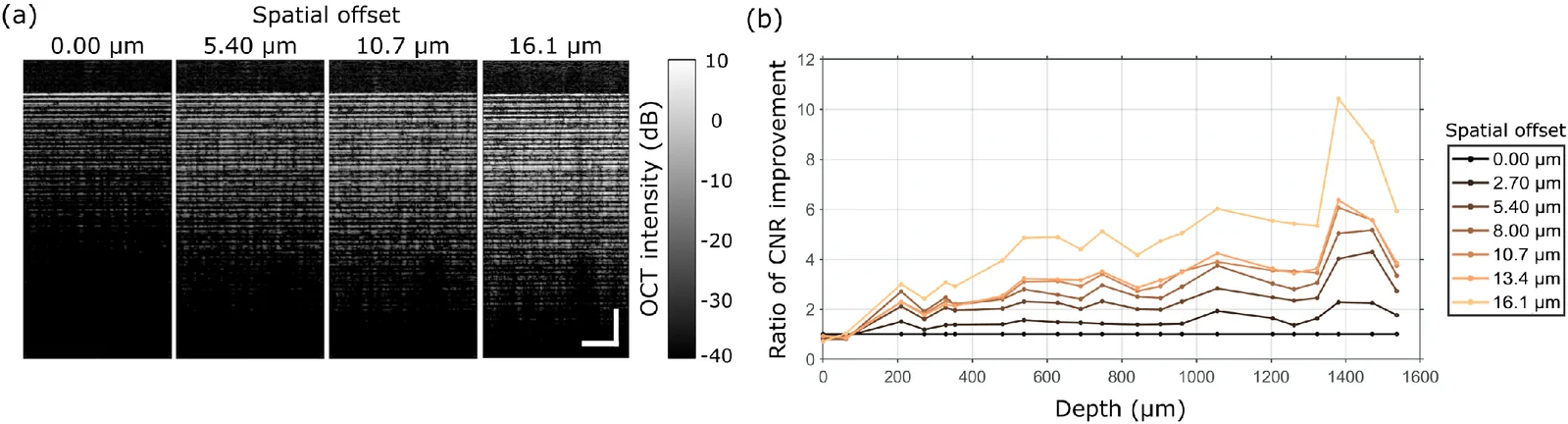
(a) SO-OCT B-scan images for layered phantom for different spatial offsets (SO = 0.00 *μ*m, 5.40 *μ*m, 10.7 *μ*m and 16.1 *μ*m). As the spatial offset is increased, layers that are otherwise not visible without the offset become evident. Scale bar: 500 *μ*m. (b) Plot indicating relative improvement in CNR at depth for different spatial offsets from figures in (a).

The evolution of CNR with depth for different spatial offsets is shown in Fig. 4(b). When evaluating the CNR for the images acquired on the layered phantom, $\mu_{\text{signal}}$ is defined as the mean signal of the adhesive layer across all A-scans, and $\sigma_{\text{signal}}$ as the corresponding standard deviation. The background terms $\mu_{\text{background}}$ and $\sigma_{\text{background}}$ are calculated from the regions between each layer, corresponding to the transparent acetate. It should be noted that there are no air gaps present between the layers; instead, the structure consists of a less scattering acetate layer sandwiched by a more highly scattering adhesive layer. Figure 4(b) shows that the ratio of CNR improvement increases with depth for all offsets, reaching a factor of 10 for the largest offset at a depth of 1,400 *μ*m. Beyond this depth, the CNR starts to degrade due to the impact of noise for long exposure times. Similar behavior was observed in a layered Parafilm phantom, shown in Supplement 1.

### 3.3. Scattering phantom

The scattering phantom was used to evaluate the performance of a homogeneous medium under more biologically relevant scattering conditions. In Fig. 5(a,b), the image acquired without offset and the merged image are shown. The merged image allows visualization of the paint layer at larger depths in the scattering material. At larger spatial offsets, some degree of axial broadening is likely to occur, which is consistent with observations from our previous manuscript [11].

**Fig. 5.**
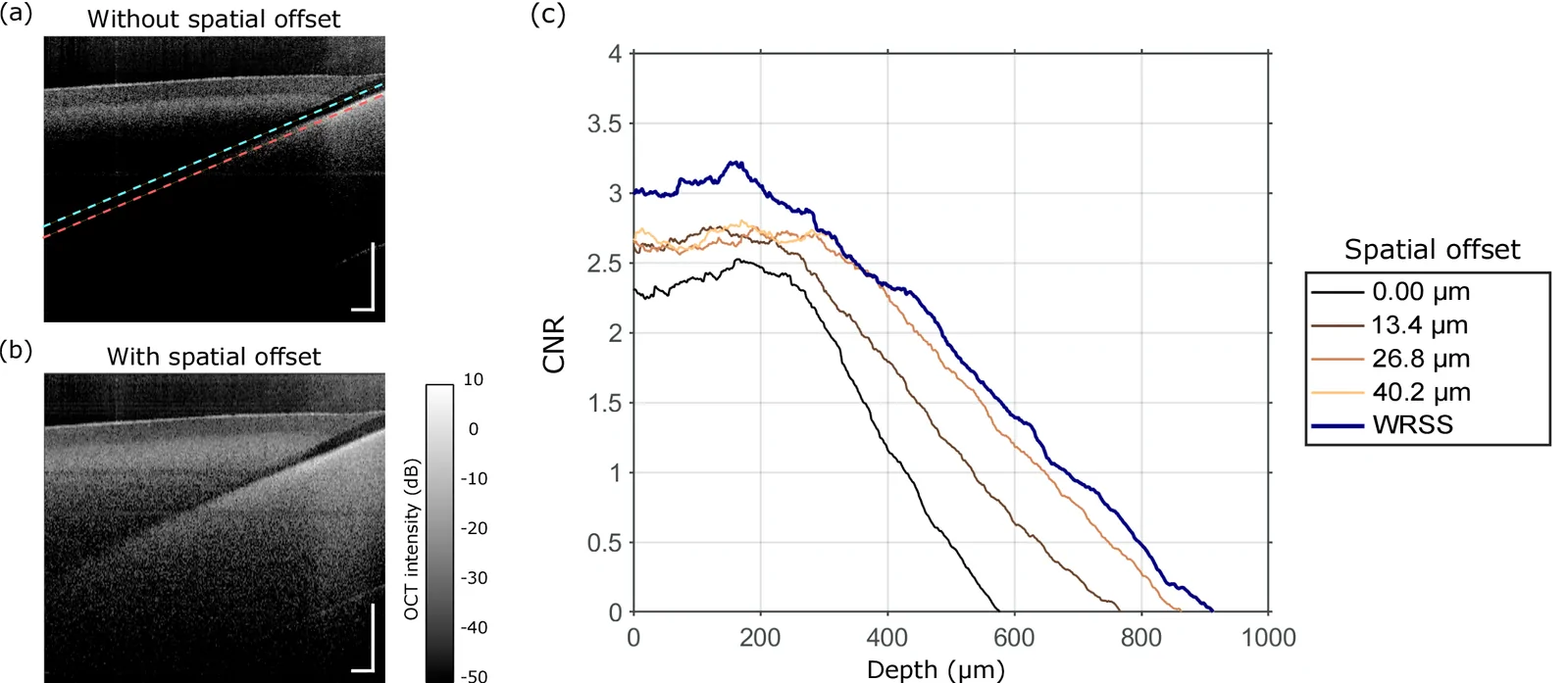
SO-OCT B-scan images of a homogeneous scattering phantom containing an angled scattering layer. (a) Image acquired without offset, and (b) weighted root-sum-square (WRSS) projection of three offset images (SO=13.4 *μ*m, 26.8 *μ*m and 40.2 *μ*m) and the zero offset image. The background material consists of butter, while the angled material is an acetate sheet (thickness = 100 *μ*m, indicated by the blue line) with white paint on its bottom surface (indicated by the red line). Scale bar: 500 *μ*m. (c) CNR plot for different offsets, with the signal term calculated from the region corresponding to the bottom painted layer of the acetate and the noise calculated from the region corresponding to the transparent acetate layer. CNR plot for the WRSS projection is shown in blue.

The CNR was calculated as a function of depth, with the signal term calculated from a vertical 10-pixel region of interest centered on the bottom painted layer of the acetate (indicated by the red line), and the noise calculated from a vertical 10-pixel region centered on the transparent acetate layer (indicated by the blue line). The resulting CNR curve was smoothed using a 300-point moving average. Figure 5(c) shows that the CNR at depth increases substantially with increasing spatial offset. The WRSS image results in an 330 *μ*m increase in penetration depth compared to the zero offset case, shown as a relative shift of the x-intercept. The CNR is maximized at a depth of 200 *μ*m, where the beam is focused. Images acquired at each spatial offset are provided in Supplement 1.

### 3.4. Biological samples

In Fig. 6, *ex vivo* imaging of the mouse eye was performed to assess the performance of SO-OCT on a sample containing strongly reflecting curved interfaces and requiring substantial imaging depth. Specular surface reflections at the corneal apex are known to produce strong surface signals and saturation artifacts in ophthalmic OCT [16,17]. An improvement in image quality at depth was observed with a corresponding CNR enhancement of 2.08 and a penetration depth of 3.6 mm was achieved in the mouse eye. The CNR improvement factor was calculated by manually selecting regions of interest corresponding to the signal and background component, followed by application of Eq. (3). Using the WRSS projection allows for visualization of the posterior lens (circled in red), while still maintaining clear imaging of the cornea. The observed flattening of the anterior chamber and the posterior lens region may be associated with tissue distortion caused by desiccation during formaldehyde fixation. To further assess deep-tissue imaging capabilities in heterogeneous biological tissues, toe and paw pad samples were investigated (Fig. 7). CNR improvements of 3.05 and 2.93 were achieved for the toe and the paw pad tissue, respectively. In both cases, the plantar surface of the hind paw was imaged. For the toe, the second digit was imaged along a trajectory extending from the claw to the digitopalmar crease, whereas for the paw pad tissue, the footpad of the fifth digit was imaged in the sagittal plane.

**Fig. 6.**
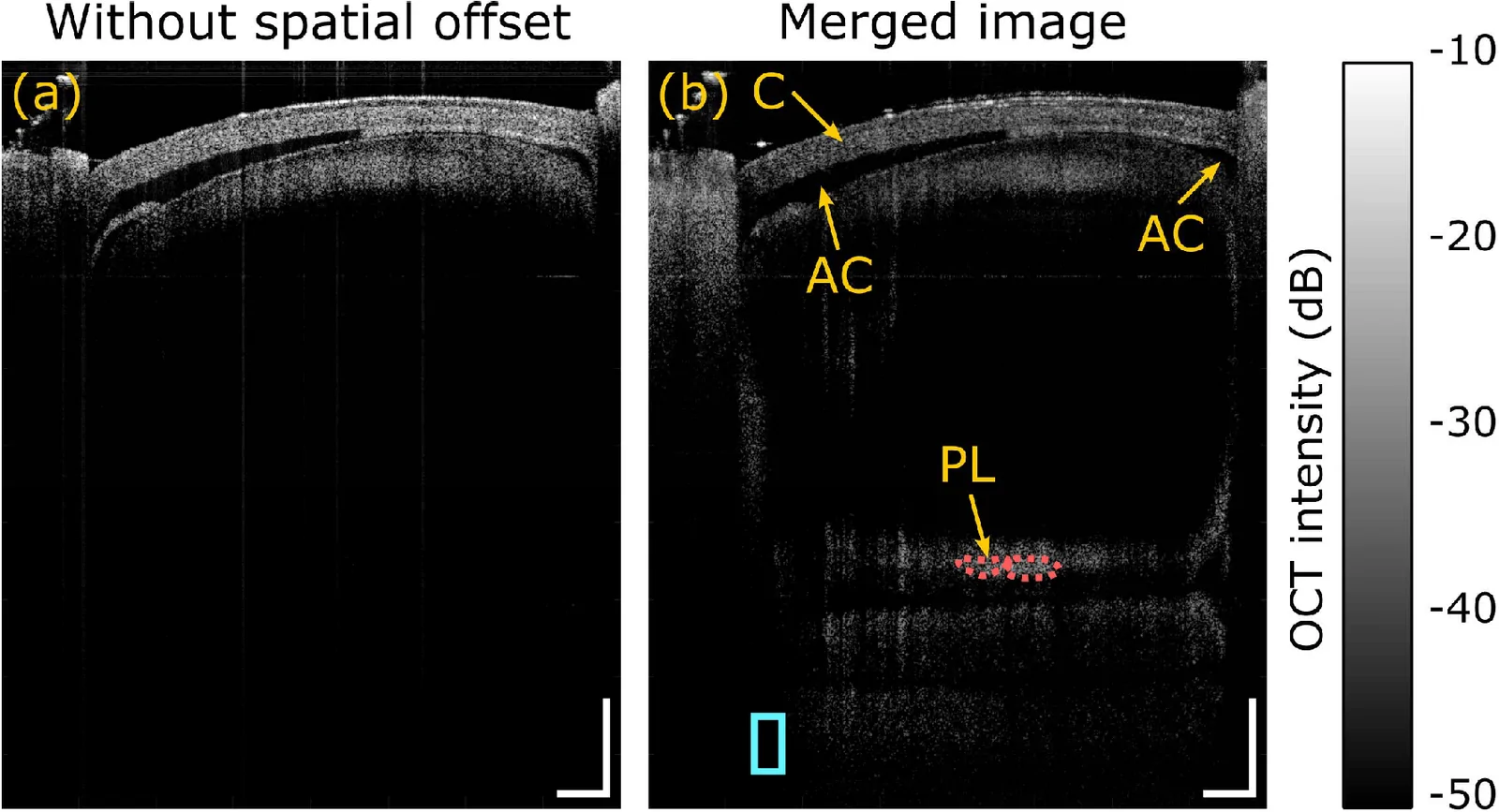
SO-OCT B-scan images of *ex vivo* mouse eye tissue. Panel (a) shows an image acquired without spatial offset, while panel (b) shows the weighted root-sum-square projection generated from the zero offset acquisition in addition to three offset acquisitions (SO = 26.8 *μ*m, 53.6 *μ*m and 80.5 *μ*m). Regions used for the CNR calculation are indicated by the red dashed line (signal) and blue solid line (background). SO-OCT imaging enables the visualization of the posterior lens (PL) surface while preserving clear delineation of the cornea (C) and anterior chamber (AC). In contrast, the posterior lens surface is not discernible in the zero-offset case. Scale bars: 500 *μ*m.

**Fig. 7.**
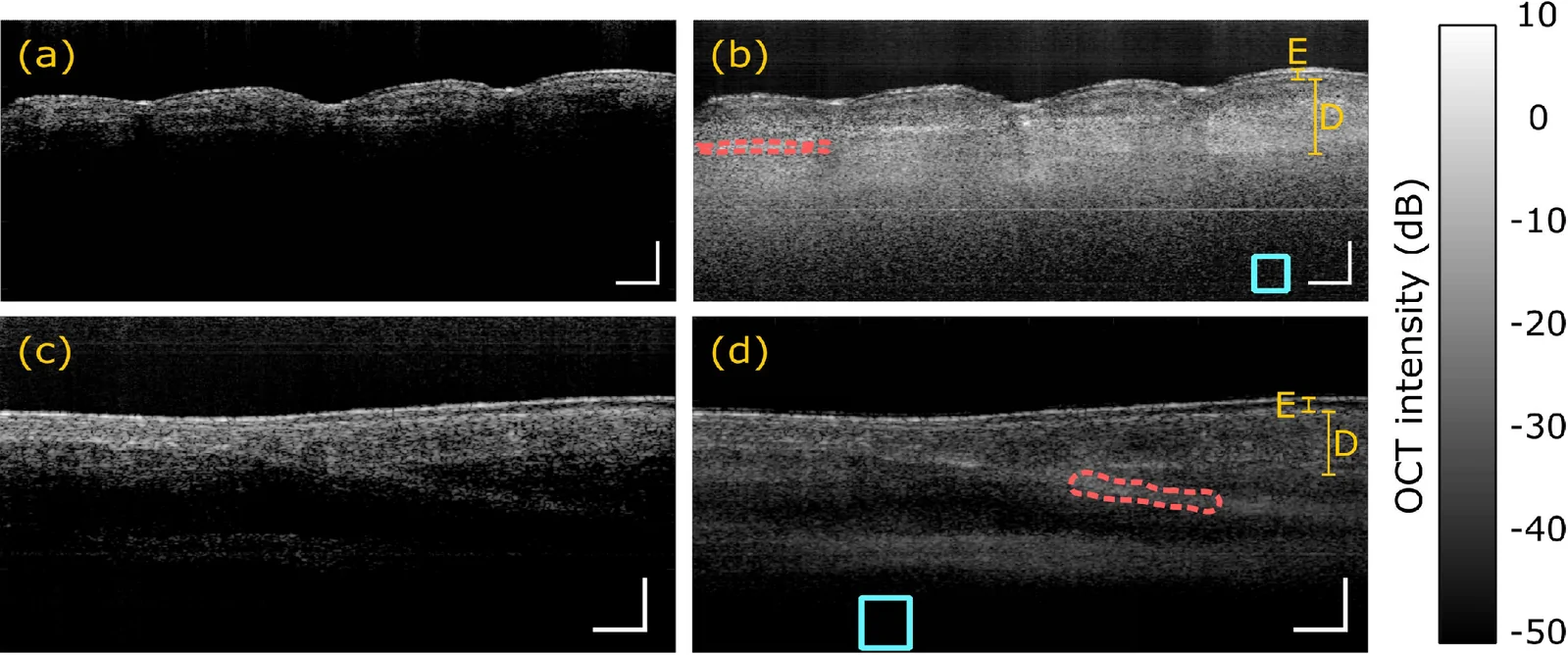
SO-OCT B-scans of *ex vivo* toe and paw pad tissue. Panels (a) and (c) show acquisitions without spatial offset, while panels (b) and (d) show weighted root-sum-square projections of toe and paw pad tissue, respectively. For toe tissues, spatial offsets of 53.6 *μ*m, 107.3 *μ*m, 160.9 *μ*m and 214.5 *μ*m were applied. For the paw pad tissue, applied spatial offsets ranged from 26.8 to 160.9 *μ*m across six evenly spaced offset values. Regions used for the CNR calculation are indicated by the red dashed lines (signal) and blue solid lines (background). Scale bars: 500 *μ*m. In the toe tissue, the dermis is clearly visible in the spatially offset image. The WRSS algorithm enhances contrast within the dermis layers (D) while preserving visualization of the epidermis (E).

In the toe tissue, the dermis was discernible only in the spatially offset image. The WRSS algorithm further enhanced contrast within the dermal layers, preserving the visibility of the epidermis. By comparison, the zero-offset image did not provide sufficient signal to distinguish either the epidermis or the dermis. In the paw pad tissue, the epidermis and dermis were only well resolved in the spatially offset case. Images acquired at each spatial offset are provided in Supplement 1.

## 4. Discussion and conclusion

In this work, we have shown that SO-OCT enables a substantial improvement in CNR at depth in both optical phantoms and *ex vivo* biological tissues. This builds upon our previous work [11], demonstrating that images acquired across multiple spatial offsets and exposure settings can be combined computationally using a WRSS algorithm, analogous to HDR photography [13], producing reconstructions with enhanced depth contrast while preserving superficial structural detail.

In the layered phantom, CNR was quantified for each layer, which increased with both spatial offset and imaging depth. The maximum CNR improvement factor of 10.4 was observed at a spatial offset of 16.1 *μ*m. This corresponds to an increase of penetration depth from approximately 2.0 mm to 3.5 mm. In the scattering phantom, SO-OCT increased penetration depth by 330 *μ*m. Finally, *ex vivo* mouse tissues were evaluated with CNR improvement factors of 2.08, 3.05 and 2.93 observed in mouse eye, toe and paw pad tissue, respectively. A penetration depth of 3.6 mm was achieved in the mouse eye.

Effective suppression of superficial surface signal and enhanced collection of signal originating from deeper regions within the sample was observed across all samples. This enabled visualization of deep structures that are otherwise obscured in zero-offset OCT due to its limited detector dynamic range. The variation in CNR improvement across tissue types, and the greater CNR improvement observed in the phantom compared to biological tissue, is attributed to differences in scattering properties and structural heterogeneity.

Related concepts of spatially separating the source and detector have been exploited in other optical imaging and sensing techniques, including spatially-offset Raman spectroscopy [18] and time-domain diffuse correlation spectroscopy [19,20]. These approaches highlight the broader potential of source-detector separation to selectively probe different sample depths, while SO-OCT applies this principle to depth-resolved structural imaging with OCT.

One advantage of SO-OCT is that it allows the focus of the illumination beam to be positioned near the surface, whilst a spatial offset allows imaging of deeper tissue layers. In the case of ophthalmology, the ability to examine the morphology of the whole eye is particularly advantageous for the ocular shape analysis for myopia [21] and detection of ocular tumors [22]. In conventional OCT, achieving whole-eye imaging typically requires focusing the beam within the eye, which compromises the effective imaging of superficial structures. Currently, whole-eye OCT is enabled with complex system changes, such as tunable lenses [23] or dual-channel OCT configurations [24]. Therefore, there is significant potential for applying SO-OCT to swept-source OCT systems, as it may enable simultaneous imaging of both the lens and the retina without altering the focus of the illumination beam. Moreover, SO-OCT only requires a simple system modification by translating a lens.

A limitation of the current SO-OCT implementation is the increased acquisition time associated with acquiring multiple spatial offsets, particularly as larger spatial offsets require longer camera exposure times to compensate for the reduced signal acquisition. At the longest exposure time used in this study (350 ms per A-scan), acquisition of the full FOV for the mouse eye image requires approximately 9.3 minutes. This acquisition time currently limits the applicability of the system for *in vivo* imaging, where motion artifacts would induce additional imaging challenges. Future work should consider potential compensation strategies such as exploring trade-offs between noise performance, illumination power, and total exposure times. Furthermore, future work could optimize the number of spatial offsets required to achieve a desired imaging depth for a given sample, thereby reducing the total number of acquisitions and measurement time. In addition, incorporating complementary contrast mechanisms, such as polarization-sensitive OCT [25] or phase-sensitive OCT [26], may further enhance the capabilities of SO-OCT in biomedical imaging.

Overall, these results highlight SO-OCT as a promising approach for enhanced depth-resolved OCT imaging for biomedical applications.

## Supplemental information

Supplement 1 Supplementary Information https://doi.org/10.6084/m9.figshare.33437350

## Data Availability

Data underlying the results presented in this paper are not publicly available at this time but may be obtained from the authors upon reasonable request.

## Supplemental document

See Supplement 1 for supporting content.
