# Supplementary material for: Spatially-offset optical coherence tomography (SO-OCT) for high dynamic range biological imaging at depth
Source: Biomed Opt Express. 2026 Sep 16;17(10):5232–42. doi: 10.1364/BOE.607448 (PMC13671703; doi:10.1364/BOE.607448)
Supplement: Supplementary file 1 [file boe-17-10-5232-s001.pdf]

Supplemental Document

Biomedical Optics EXPRESS

Spatially-offset optical coherence tomography (SO-OCT) for high dynamic range biological imaging at depth: supplement

EMI C. V. HUGHES,[1,*] RALF MOUTHAAN,[1] FREJA HØIER,[2] GAVRIELLE R. UNTRACHT,[2] PETER E. ANDERSEN,[2] AND KISHAN DHOLAKIA[1,3]

[1] *Centre of Light for Life, School of Biological Sciences, Adelaide University, Adelaide, SA 5005, Australia*
[2] *Department of Health Technology, Technical University of Denmark, Ørsteds Pl. 345C, 2800 Kongens Lyngby, Denmark*
[3] *SUPA, School of Physics and Astronomy, University of St Andrews, St Andrews KY16 9SS, UK*
[*] *emi.hughes@adelaide.edu.au*

This supplement published with Optica Publishing Group on 16 September 2026 by The Authors under the terms of the Creative Commons Attribution 4.0 License in the format provided by the authors and unedited. Further distribution of this work must maintain attribution to the author(s) and the published article's title, journal citation, and DOI.

Supplement DOI: https://doi.org/10.6084/m9.figshare.33437350

Parent Article DOI: https://doi.org/10.1364/BOE.607448

Spatially-offset optical coherence tomography (SO-OCT) for high dynamic range biological imaging at depth: supplemental document

1 Preparation of phantoms

1.1 Layered phantom

The layered phantom consists of a stacked microscope spacer (Sigma Aldrich, thickness 120 μm) and comprising 20 layers mounted on top of a glass microscope slide with no gaps between the layers. The strong optical reflectivity between the acetate and adhesive layers simulates discrete reflective boundaries and enables evaluation of CNR between the layers.

1.2 Scattering phantom

The scattering phantom was prepared using commercially available butter to provide a nominally homogeneous scattering medium. The butter was poured into a custom mold, and the acetate was embedded within the butter to introduce a depth-dependent signal reduction. The underside of the acetate was coated with a thin, uniform layer of white paint to enhance the back-scattering contrast.

2 SO-OCT on layered Parafilm phantom

SO-OCT images with matched surface intensities for the layered Parafilm phantom are shown in Fig. 1. Similarly to the microscope spacer results presented in Fig. 4(a) of the main text, layers otherwise not visible become apparent with increasing spatial offset.

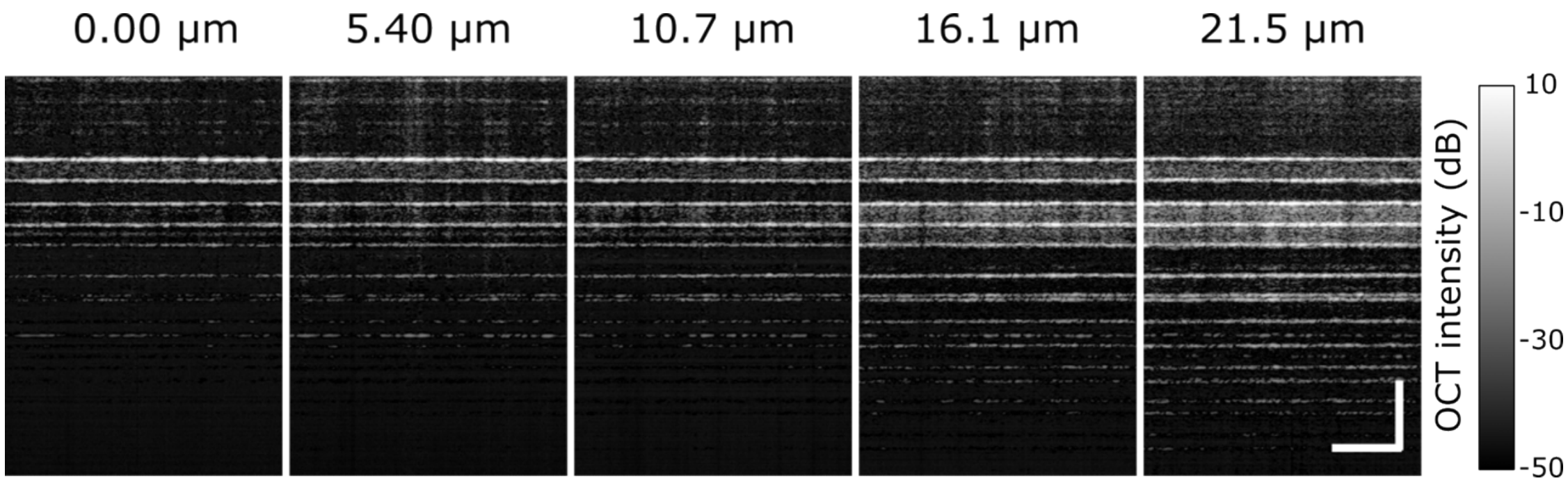

SO-OCT B-scan images of the layered Parafilm phantom acquired at different spatial offsets (scale bar: 500 μm). Increasing the spatial offset reveals deeper layers that are not readily visible at smaller spatial offsets.

3 SO-OCT on scattering phantom

SO-OCT images with matched surface intensities for the scattering phantom are shown in Fig. 2. Increasing the spatial offset improves visualization of the paint layer at depth that is otherwise not visible with smaller offsets. In contrast, signals originating directly beneath the surface of the butter layer are progressively attenuated as the offset increases.

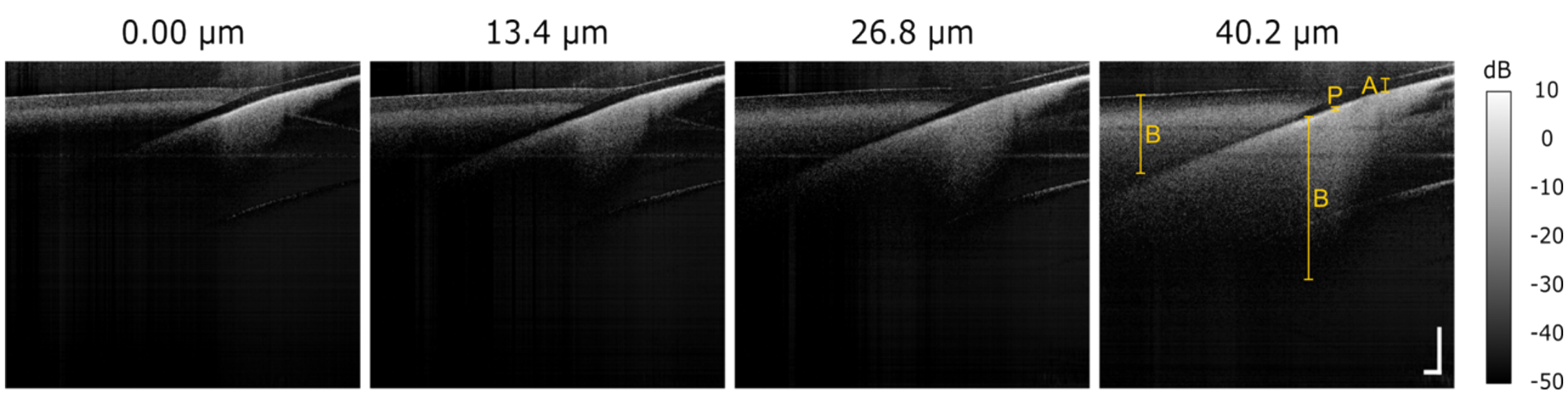

SO-OCT B-scan images for the scattering phantom for different spatial offsets (scale bar: 500 μm). The scattering phantom consists of butter (B), diagonally embedded with acetate (A), the lower surface of which is coated with a layer of white paint (P).

4 SO-OCT on mouse eye tissue

SO-OCT images with matched surface intensities for the mouse eye are shown in Fig. 3. The largest spatial offset at 80.5 μm enables visualization of posterior lens surface, which is not visible with the 0.0 μm offset image. In contrast the 0.0 μm image provides the clearest visualization of the cornea and anterior chamber.

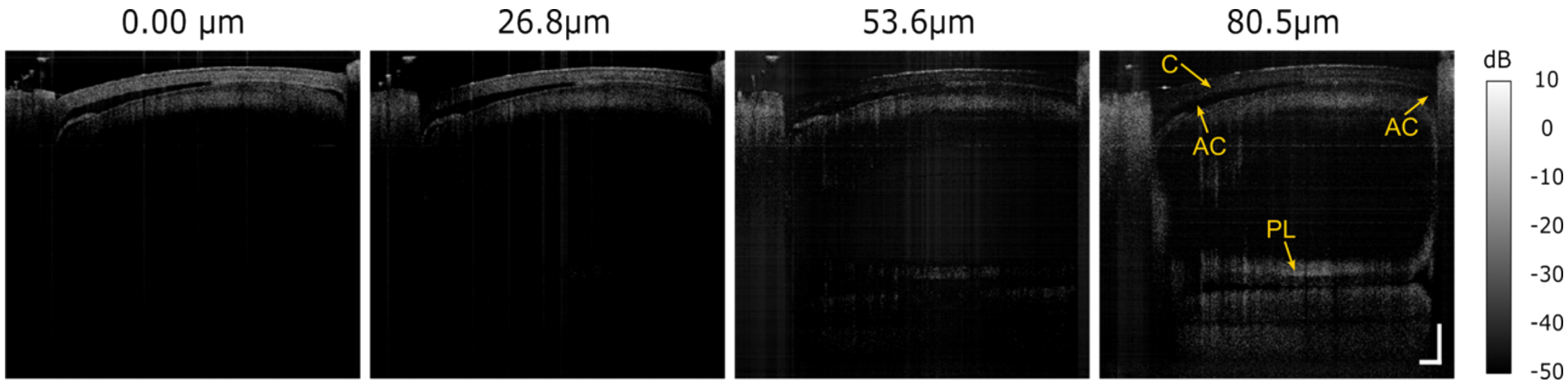

SO-OCT B-scan images of the mouse eye acquired at different spatial offsets (scale bar: 500 μm). The labels indicate the posterior lens (PL), cornea (C) and anterior chamber (AC).

5 SO-OCT on mouse toe tissue

SO-OCT images with matched surface intensities for the mouse toe are shown in Fig. 4. Larger spatial offsets enable visualization of deeper tissue structures, such as the dermis, however, minimal additional signal is observed beyond an offset of 160.9 μm. Among all investigated samples, the mouse toe tissue exhibits the most pronounced reduction in subsurface signal intensity of the epidermis with increasing offset.

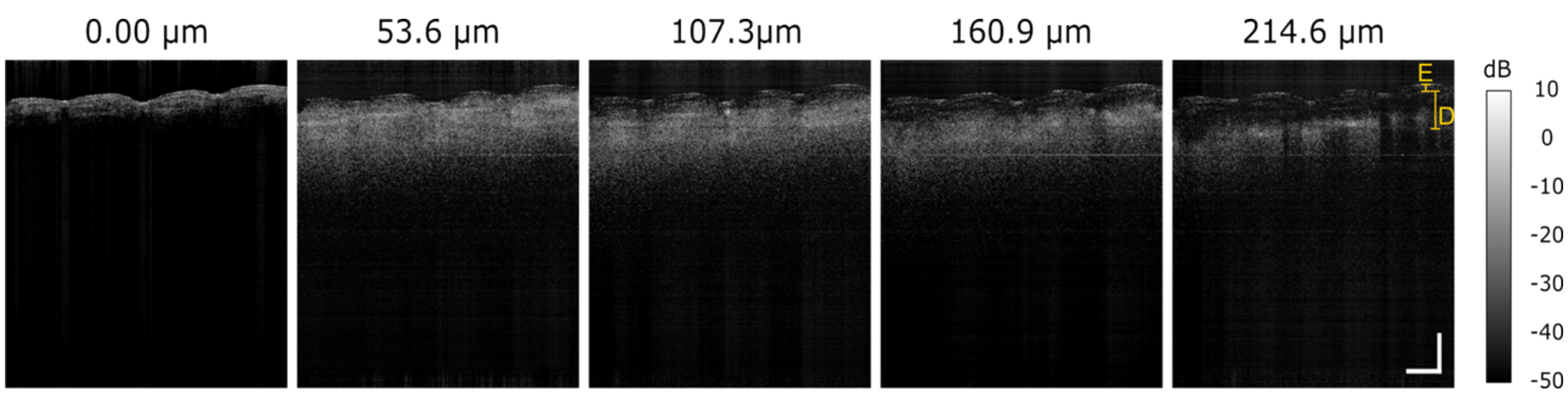

SO-OCT B-scan images for mouse toe for different spatial offsets (scale bar: 500 μm). The labels indicate the epidermal layers (E) and dermis (D).

6 SO-OCT on Mouse Paw

SO-OCT images with matched surface intensities are shown for the mouse paw in Fig. 5. The epidermis is only observable for images acquired at spatial offsets larger than 26.8 μm.

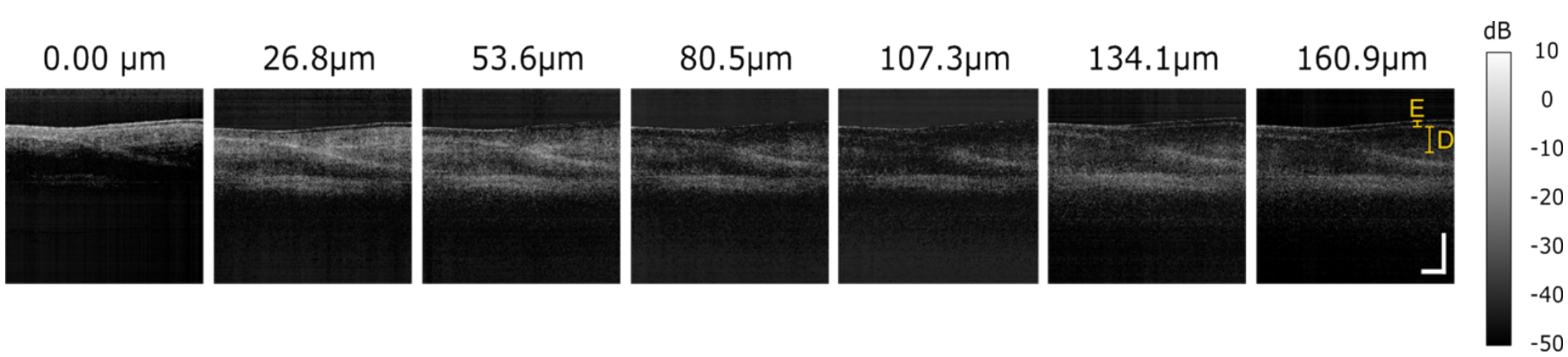

SO-OCT B-scan images for mouse paw for different spatial offsets (scale bar: 500 μm). The labels indicate the epidermal layers (E) and dermis (D).

7 Comparison of weighted-sum and weighted root-sum-square (WRSS) image merging algorithms

In Fig. 6, the WRSS approach is compared with the weighted sum image merging method. The WRSS approach shows greater contrast in superficial layers compared to the weighted sum image in both the mouse paw pad and mouse toe images.

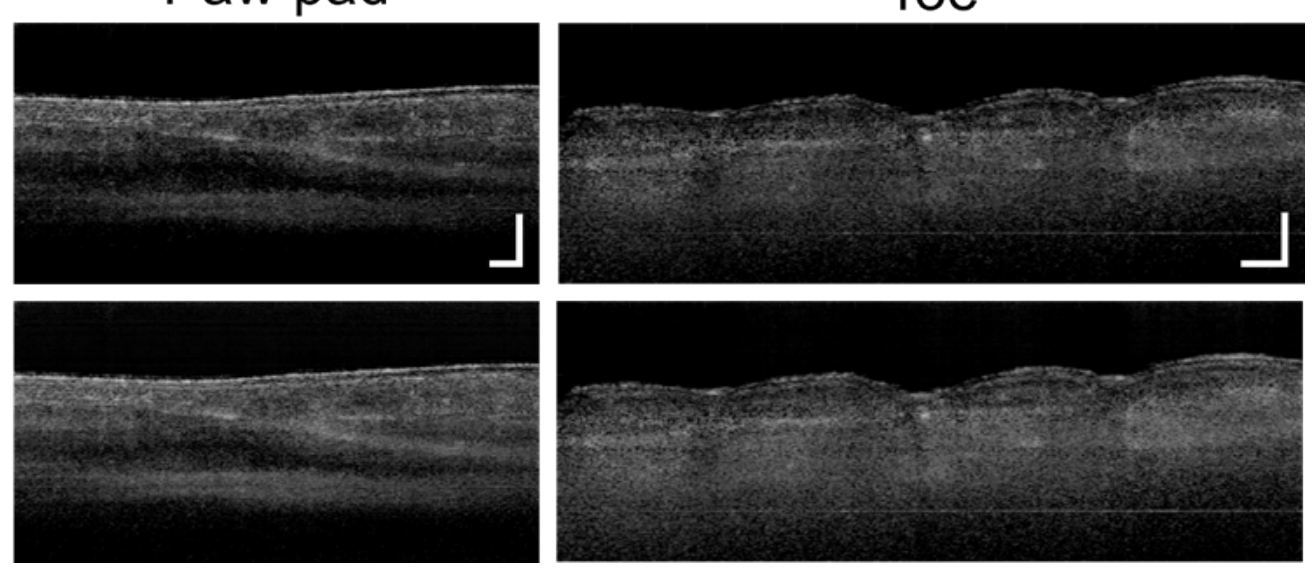

Comparison of weighted-sum and weighted root-sum-square (WRSS) image merging methods applied to SO-OCT B-scans for mouse paw pad and toe tissue. Scale bar: 250 μm).

8 Camera exposure parameters

Increasing the camera exposure time enables better utilization of the camera dynamic range to detect signal originating from deeper within the sample. In comparison, numerical normalization in post-processing will only rescale the measured signal, hence no advantage of acquisition with a large spatial offset would be observed. The exposure time was manually tuned to remain just below saturation, such that the detected sample arm signal could be matched to the reference arm power. To rapidly estimate the average surface OCT signal across the FOV, a spatially undersampled set of A-scans is acquired using an A-scan undersampling factor of 10, corresponding to a tenfold reduction in the number of A-scans acquired over a given ROI. Each interferogram was converted from wavelength to wavenumber using $k = 2\pi/\lambda$, interpolated onto a uniformly spaced wavenumber axis, and reconstructed using an inverse fast Fourier transform of the interferogram, enabling rapid averaging of the surface OCT signal. This process was performed iteratively for all spatial offsets until the normalization error was reduced to below 1% relative to the maximum offset. Increasing the camera exposure time enables better utilization of the camera dynamic range to detect signal originating from deeper within the sample. In comparison, numerical normalization in post-processing will only rescale the measured signal, hence no advantage of acquisition with a large spatial offset would be observed. Tables 1 and 2 show the exposure time settings used for phantom and biological samples, respectively.

The differences in offset/exposure combinations between samples in Tables 1 and 2 reflect differences in sample morphology, scattering, and surface reflection, which resulted in different contrast performance and exposure requirements.

Exposure times (ms) used for phantom samples at different spatial offsets (μm)

**Sample**	**Spatial offset (μ m)**	**Exposure time (ms)**
Layered phantom	0.00	1
	2.68	3
	5.37	7
	8.05	10
	10.7	13
	13.4	15
	16.1	32
Scattering phantom	0.00	30
	13.4	85
	18.8	85
	21.5	245
Parafilm	0.00	8
	2.68	8
	5.37	13
	8.05	14
	10.7	18
	13.4	32
	16.1	57
	18.8	70
	21.5	120

Exposure times (ms) used for biological samples at different spatial offsets (μm)

**Sample**	**Spatial offset (μ m)**	**Exposure time (ms)**
Mouse eye	0.0	25
	26.8	45
	53.6	150
	80.5	350
Mouse toe	0.0	12
	53.6	250
	107.3	275
	160.9	300

Sample	Spatial offset (μ m)	Exposure time (ms)
	214.6	350
Mouse paw	0.0	37
	26.8	180
	53.6	250
	80.5	195
	107.3	200
	134.1	350
	160.9	330
